# Supplementary material for: Sustained-input switches for transcription factors and microRNAs are central building blocks of eukaryotic gene circuits
Source: Genome Biol. 2013 Aug 23;14(8):R85. doi: 10.1186/gb-2013-14-8-r85 (PMC4054853; doi:10.1186/gb-2013-14-8-r85)
Supplement: Additional file 5 — HTML Browsable Motif Output. Zipped folder containing all WaRSwap and FANMOD motif output, viewable in a web browser. [file gb-2013-14-8-r85-S5.ZIP › HTML_browsable_motif_output/WaRSwap_all_tair9/sigs_WSWRSLDraw-2000_all.pvals.heatmaps.html/motif_id_164_010100100_tftype_all_upstream_-1000_0.html]

```
BG_MODEL = Rnew2
MOTIF_ID = 164_010100100
TF_TYPE = all
UPSTREAM = -1000_0


PVals
FN_0.2	FN_0.4	FN_0.6	FN_0.8
dg_60.genes	0.004	0.135	0.628	0.688
dg_70.genes	0.003	0.135	0.651	0.669
dg_80.genes	0.005	0.145	0.646	0.675

ZScores
FN_0.2	FN_0.4	FN_0.6	FN_0.8
dg_60.genes	2.686	1.108	-0.395	-0.589
dg_70.genes	2.777	1.101	-0.436	-0.559
dg_80.genes	2.7	1.093	-0.426	-0.578

StDevs
FN_0.2	FN_0.4	FN_0.6	FN_0.8
dg_60.genes	460.103	546.088	261.582	52.64
dg_70.genes	448.817	549.902	276.142	51.101
dg_80.genes	457.405	553.82	275.059	50.392
```
